# Almond Supplementation Improves Acne Lesions and Skin Microbial Diversity in Adults with Mild to Moderate Acne Vulgaris

**DOI:** 10.3390/nu18040625

**Published:** 2026-02-13

**Authors:** Panchali Moitra, Jagmeet Madan, Krisha Shah, Pradnya Mandavkar, Rajiv Joshi, Soumik Kalita, Shobha A. Udipi

**Affiliations:** 1 Department of Postgraduate Programs and Research, Sir Vithaldas Thackersey College of Home Science (Empowered Autonomous Status), Shreemathi Nathibai Damodar Thackersey Women’s University, Mumbai 400049, India; 2 Department of Food, Nutrition and Dietetics, Sir Vithaldas Thackersey College of Home Science (Empowered Autonomous Status), Shreemathi Nathibai Damodar Thackersey Women’s University, Mumbai 400049, India; 3 Research, Innovation, Consultancy & Collaboration Centre, Sir Vithaldas Thackersey College of Home Science (Empowered Autonomous Status), Shreemathi Nathibai Damodar Thackersey Women’s University, Mumbai 400049, India; 4 Clinical Aesthetics and Investigative Management Services Pvt Ltd., Andheri East, Mumbai 400093, India; 5 FamPhy, Gurugram 122101, India; soumik.kalita@famphy.com; 6 Kasturba Integrative Health Society—Medical Research Foundation (KiHS-MRF), Vile Parle West, Mumbai 400056, India

**Keywords:** acne diet, acne vulgaris and nuts, acne in young adults, almonds and acne, diet and skin health, India, microbiome and skin health, nuts and skin health, nutrients and dermatology, skin microflora and nutrition

## Abstract

Objectives: This randomized, controlled, parallel-group study was conducted to evaluate the effectiveness of daily almond consumption on acne lesion counts, skin hydration, sebum production, and skin microflora composition in 18–35-year-old young adults with acne vulgaris in Mumbai, India. Methods: A defined amount of whole, unsalted almonds with skin (60 g) was provided to the experimental group (n = 36). The control group (n = 38) received isocaloric cereal-pulse-based snack varieties. The primary endpoints were changes in inflammatory, non-inflammatory, and total acne lesion counts after 20 weeks of supplementation. Secondary endpoints included changes in facial sebum, hydration levels, skin morphology and microflora, and selected biochemical parameters. Results: At week 20, the almond group showed greater reductions in total lesion counts (−22.2% vs. −9.8%), inflammatory lesion counts (−8.3% vs. +12%), and non-inflammatory lesion counts (−26.1% vs. −20.4%) than controls. Objective lesion volume, area, and height measures for both single and clustered acne decreased in the almond group (*p* ≤ 0.001). Microbial diversity increased, with the Shannon index (2.6 to 3.4 (*p* = 0.039) and the Chao1 richness index (266.9 → 835.2; *p* < 0.001) showing improvements at endline. Moreover, significant post-intervention changes in the psychosocial outcomes, such as the acne-related quality of life scores (*p* < 0.001) and anxiety symptoms (*p* = 0.016), were observed in the almond group. Conclusions: Daily almond consumption reduced acne lesion count and improved skin microbial diversity and acne-specific quality of life, highlighting its potential to complement standard acne treatments and support skin health.

## 1. Introduction

Acne vulgaris is one of the most common dermatological conditions with peak prevalence in youth and a well-documented negative impact on quality of life [1,2]. As a multifactorial inflammatory skin condition, acne results from increased sebum production, abnormal follicular keratinization, proliferation of *Cutibacterium acnes* bacteria, and a host of inflammatory responses within the pilosebaceous unit in the skin [2,3,4]. Although the pathogenesis of acne involves an interplay of genetic, hormonal, environmental, and lifestyle factors, the role of diet and nutrition in modulating the development and severity of acne has received considerable attention in recent years [5,6]. Emerging evidence implicates systemic metabolic and hormonal signals, notably insulin and insulin-like growth factor-1 and nutrient-sensitive pathways, such as mTORC1, in modulating sebocyte lipogenesis, keratinocyte proliferation, and inflammatory mediator activation that collectively contribute to acne lesion formation [4,7,8,9]. These mechanistic links provide biological plausibility for the modulation of acne through dietary exposures, particularly those that influence glycemia, insulin/IGF-1 signaling, and systemic inflammation.

Previous food-based studies for acne have ranged from macronutrient manipulation (low glycemic load), dairy restriction, and omega-3 supplementation to trials of specific micronutrients or botanical extracts with anti-inflammatory or improved skin barrier function and biophysical properties [10,11,12,13]. Observational studies have reported associations between plant-based foods and better skin health [5,14] and dairy intake and higher acne prevalence [11] and also explored various dietary triggers of acne flare ups [5,7,10,15]. Systematic reviews and meta-analyses have noted high-glycemic-load foods to influence sebum production [10,11,16], and randomized trials have reported clinical improvements in inflammatory pathways linked to acne development, following adoption of low-glycemic-load diets [9,13,17]. Other dietary factors, such as probiotics, polyphenols, and Mediterranean dietary patterns, have also been investigated to influence systemic inflammation [18,19,20] and the gut microbiome pathways that may secondarily affect skin microflora composition and cutaneous immune responses [21,22]. Collectively, this growing body of evidence demonstrates the potential of food-based interventions as adjunctive strategies for acne management, highlighting the need to develop targeted intervention studies that further investigate the association between dietary factors and acne and measure both clinical and intermediate biological endpoints.

Almonds are nutrient-dense tree nuts rich in monounsaturated fatty acids, vitamin E, fiber, micronutrients such as magnesium and zinc, and polyphenolic compounds that are known for their antioxidant and anti-inflammatory properties [23,24,25]. These properties are particularly relevant to acne, a condition driven by oxidative stress, inflammation, and alterations in sebum production. Moreover, almonds have been shown to improve postprandial glycemia, lipid metabolism, and overall cardiometabolic profiles [25,26], suggesting potential downstream effects on pathways that regulate cutaneous inflammation, epidermal barrier function, and the ecological balance of skin microflora, thereby influencing acne pathogenesis and clinical outcomes.

Despite plausible mechanisms, research examining almonds in relation to acne outcomes is limited. To date, most clinical research on almonds and skin has evaluated aging-related endpoints, such as wrinkle severity [27,28]. Rigorous experimental studies that investigate the influence of almond intake on acne and skin microbiome profiling are needed to determine whether almonds exert clinically meaningful effects in acne. In this context, the current study was conducted to evaluate the effectiveness of daily almond consumption on acne lesion counts, skin hydration, sebum production, and skin microflora composition in 18–35-year-old young adults with acne vulgaris in Mumbai, India. We anticipate that the findings of this research will offer valuable insights into non-invasive, food-based strategies for acne management with the added potential to confer broader cardiometabolic and nutritional benefits beyond improvements in skin health outcomes.

## 2. Materials and Methods

### 2.1. Study Design and Participants

This randomized, controlled, parallel-group, single-blind intervention study was conducted among men and women aged 18–35 years with mild to moderate facial acne vulgaris in Mumbai, India. Participants were recruited from the community through study advertisements disseminated via flyers and notices placed in residential complexes, educational institutes, and community centers between January and May 2024. Individuals who expressed interest were contacted by the study team, and initial screening visits were scheduled at dermatologist’s clinic to assess eligibility for participation.

Clinical grading of acne was performed by an experienced dermatologist using the Investigator Global Assessment (IGA) scale, a standardized clinician-reported measure of acne severity [29,30]. Participants with mild to moderate acne (IGA scores 2–3) who had been stable on their topical or oral acne regimen, except if the medication was a noted exclusion, for at least four weeks were eligible for inclusion in the trial. Individuals were excluded if they had minimal (IGA scores 0–1) or severe (IGA score 4) facial acne, a known nut allergy, were receiving isotretinoin therapy, had used oral antibiotics or any oral medications for acne in the preceding three months, and/or had taken oral probiotic or vitamin E supplements within one month prior to recruitment. Written informed consent was obtained from all participants prior to enrolment.

### 2.2. Intervention Protocol

Eligible participants were randomized to either the almond group or the control group using a computer-generated block randomization procedure, ensuring balanced allocation by sex. A defined amount of whole, unsalted almonds with skin (60 g), calculated to provide approximately 20% of the total daily energy intake (estimated as 1800–2000 Kcal/day, as per previous studies [31,32,33] conducted in this age group in urban India), was provided to the experimental group in two divided doses at midmorning and evening/midafternoon under supervision. The control group received cereal-pulse-based snack varieties, developed and standardized to be isocaloric to 60 g of almonds or approximately 350–360 Kcal. Given that fiber, fatty acid composition, and micronutrients, such as zinc or vitamin E, are integral components of the almond food matrix and represent potential mechanistic pathways through which almonds may exert their metabolic effects on skin and overall health, the nutritional profiles of control snacks were not matched to those of almonds.

The almond and control food packets were delivered to participants on a fortnightly basis through door-to-door distribution. Compliance with product intake was monitored by supervising consumption during daily video calls. Adherence was defined as consuming more than 80% of the assigned product over the 20-week intervention period. Participants were asked to follow their usual dietary patterns and avoid consumption of any other tree nuts or products containing nuts during the trial period.

The primary endpoints of the study were changes in inflammatory (papules, pustules, and nodules), non-inflammatory (open and closed comedones), and total lesion counts (sum of inflammatory lesions and non-inflammatory lesions) after 20 weeks of supplementation. Secondary endpoints included changes in other skin-related investigations, such as sebum, hydration, and 3D skin imaging (to determine volume, conforming area, and maximum height of single and cluster acne), skin microflora, and selected biochemical parameters.

### 2.3. Sample Size

**Figure 1:**
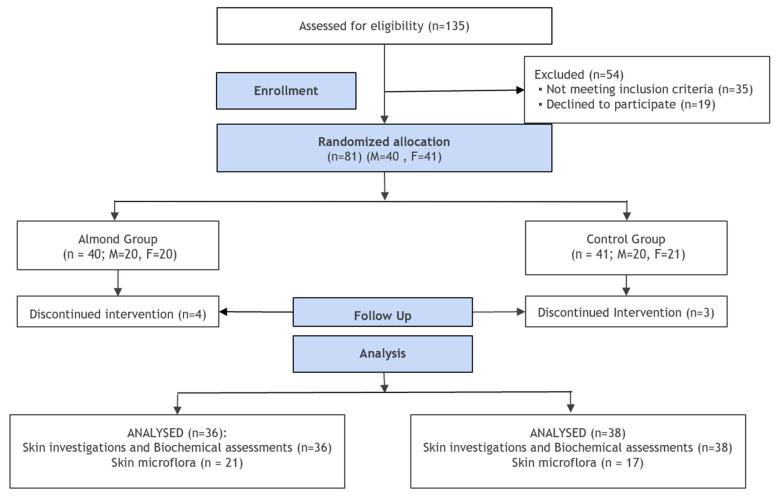
CONSORT diagram of participant screening, randomization, and final analysis.

Sample size calculations were based on detecting a clinically meaningful 15–25% reduction in acne lesion counts, consistent with improvements reported in prior nutritional and dietary intervention trials [13,17]. Using a two-tailed α = 0.05, 80% power, and a 95% confidence interval, the minimum required sample size was estimated to be 30 participants per group. To account for an anticipated 20% dropout, the target enrolment was increased to 36 participants per arm (total n = 72). Given that participant recruitment was done in community settings, we assumed the prevalence of acne as 55–65%, as reported in recent epidemiological studies in India [1,2,3]. Accordingly, 130–150 individuals were planned to be screened to obtain the desired sample. The detailed sampling and recruitment protocol is presented in Figure 1.

### 2.4. Outcomes

#### 2.4.1. Acne Count

Clinical grading and lesion counting were performed by a qualified dermatologist blinded to group allocation using the IGA scale [30] and a modified Cunliffe–Leeds technique [29], respectively. To ensure accurate identification and grading of all lesions, a transparent grid-marked acetate sheet was gently placed on the facial surface using anatomical landmarks, such as the ears, chin, and nasal tip, for consistent positioning. Each facial side was assessed separately, and lesions were palpated when necessary to confirm lesion type. All dermatological assessments were conducted by the same physician to ensure procedural consistency and reproducibility.

#### 2.4.2. Sebum and Hydration Assessments

At week 0 (baseline) and at follow-up visits during weeks 5, 10, 15, and 20, facial sebum content was measured using the SebumScale (Delfin Technologies Ltd., Kuopio, Finland), a compact device that utilizes quartz crystal microbalance technology to provide accurate and repeatable measurements in micrograms per square centimeter. After this assessment, participants washed their faces with a dermatologist-approved noncomedogenic cleanser and were then seated in an acclimatization room maintained at controlled environmental conditions (22–24 °C and 40–60% relative humidity) for 30–45 min before further evaluations. Skin surface hydration (stratum corneum) was assessed using the MoistureMeter SC (Delfin Technologies Ltd., Kuopio, Finland). Values < 20 indicated dry skin, values between 20 and 40 were considered normal, and values > 40 indicated well-hydrated skin [34,35,36].

The Antera^®^ 3D (Miravex Ltd., Dublin, Ireland) system was used for image acquisition and analysis of acne lesion volume, conforming area, and maximum height. This device employs multi-directional illumination and computer-aided surface reconstruction to quantify skin topography and chromophore distribution. Images are generated by illuminating the skin with LEDs of varying wavelengths (455–625 nm) and performing spatial and spectral analysis to produce high-resolution, three-dimensional surface reconstructions for quantitative evaluation. Additionally, standardized facial photographs were obtained using the C-Bright photographic setup, which integrates controlled LED illumination and fixed positioning to generate reproducible clinical images. A laser alignment guide ensured consistent facial positioning across visits, while uniform LED lighting minimized shadows and maintained consistent color. Photographs were captured from three angles—left profile (0°), front (90°), and right profile (180°)—at each visit and subsequently transferred to a computer for analysis and documentation.

#### 2.4.3. Skin Microflora

Skin microflora was sampled at week 0 and week 20 in a subsample of 38 participants (almond group (n = 21) and control group n = 17)) by swabbing standardized facial regions with sterile swabs followed by DNA extraction and sequencing at an NABL-accredited genetic testing laboratory to estimate microbial relative abundances. The samples were extracted using a QIAamp BiOstic Bacteremia DNA Kit (QIAGEN, Hilden, Germany) (Cat# 12240-50) and quantified using a Qubit DNA High sensitivity Assay (Invitrogen, Thermo Fisher Scientific (Waltham, MA, USA), Cat# Q32854). The PCR products were further taken for DNA library preparation using a Twist MF Library Prep Kit for Illumina (Twist Bioscience, Cat#_100876, South San Francisco, CA, USA). Amplicon sequencing of the V1-V2 region of 16S rRNA gene was performed using Illumina NextSeq platform (Illumina, San Diego, CA, USA). A quality check on the raw fastq files was done using the FASTQC toolkit to check for base quality, base composition, and GC content. Forward and reverse reads were stitched together using a join pairs module of QIIME 2 with default parameters. Chimeric sequences were filtered out using the uchime utility from VSEARCH using a de novo approach, and taxonomy classification was done at phyla, order, family, genera, and species levels. To monitor background contamination, extraction blanks and PCR negative controls were processed and sequenced in the same experimental runs as the study samples. The sequencing depth per sample ranged from 115,285 to 7,122,846 paired-end reads, ensuring sufficient coverage for robust taxonomic profiling and diversity analyses. For quality control, a stringent filtering threshold was applied using a Phred quality score of 30, corresponding to 99.9% base-calling accuracy. Reads not meeting this threshold were trimmed or excluded prior to downstream analysis to minimize sequencing errors and improve the reliability of taxonomic assignment and diversity metrics.

Microbial diversity was quantified using the Shannon diversity index (H), calculated as H = −Σ (pi ln pi), where pi represents the proportional abundance of each taxa within the microbial community [37]. To estimate the richness of the microbial community or the total number of different species (OTUs) present, we calculated the Chao1 index. Higher Chao1 values indicate greater species richness [37]. Additionally, the total number of genera was determined by assigning all quality-filtered 16S rRNA sequencing reads to taxonomic units and counting the unique genera detected in each sample at baseline and endline to assess post-intervention changes.

#### 2.4.4. Biochemical Assessments

Venous blood samples were taken in the fasted state at week 0 (baseline), week 10 (midline), and week 20 (endline) visits. The total cholesterol was estimated using the CHOD-PAP (Cholesterol Oxidase–Phenol Aminophenazone) method, triglycerides using the GOD-POD (Glucose Oxidase–Peroxidase) method, and HDL cholesterol using the immunoinhibition and CHO-POD (Cholesterol Oxidase–Peroxidase) method. Fasting glucose was estimated using the hexokinase method, fasting insulin using the the immunoenzymatic method, thyroid profile using the the CLIA (Chemiluminescence Immunoassay) method, and morning cortisol and high sensitivity CRP using the the immunoenzymatic method. Inflammatory markers, such as Tumor necrosis factor-alpha (TNF-alpha) and the interleukin-6 (IL-6), were analyzed using the ELISA method using the Elabscience^®^ kit (Elabscience Biotechnology, Wuhan, China).

#### 2.4.5. Anthropometry, Stress, Sleep, and Diet

Anthropometry, diet, and lifestyle-related covariates were assessed at baseline and endline in almond and control groups. Weight and body composition were assessed using the Tanita^®^ DC-240 Body Composition Analyzer (Tanita, Arlington Heights, IL, USA), and height was measured using a stadiometer. Anxiety and stress symptoms were evaluated using the Generalized Anxiety Disorder-7 (GAD-7) [38] and the Perceived Stress Scale-10 (PSS-10) [39], respectively. Sleep quality was assessed with the Pittsburgh Sleep Quality Index (PSQI), with a PSQI score ≥ 5 indicating poor sleep quality [40,41]. Three consecutive-day, multiple-pass, 24 h dietary recalls were conducted by trained nutritionists at weeks 0, 5, 10, 15, and 20 to estimate energy intake. The dietary recall data were analyzed using the dietary assessment software DietCal Version 7.0 (Profound Tech Solutions, New Delhi, India) [42].

#### 2.4.6. Acne-Related Quality of Life

Acne-related quality of life was assessed using the Acne Life Quality Index (ALQI), a 10-item validated questionnaire that evaluates self-perception and appearance concerns, emotional and social functioning, and perceived symptom and treatment burden of acne, with total scores ranging from 0 to 30 [43]. Higher scores indicate greater impairment in quality of life, reflecting more emotional distress, social discomfort, and functional limitations due to acne.

### 2.5. Statistical Analysis

Statistical analyses were performed using Jamovi^®^ (version 2.6.17; The Jamovi Project, Sydney, Australia) [44]. Data distributions were examined using the Shapiro–Wilk test and visual inspection of Q–Q plots. As most variables deviated from normality (*p* < 0.05), nonparametric tests were employed. Continuous variables are presented as medians with interquartile ranges (IQRs), and categorical variables as frequencies and percentages. Within-group changes across study visits were assessed using the Friedman test, with post hoc pairwise comparisons conducted using Wilcoxon signed-rank tests; effect sizes were quantified using rank biserial correlations. Between-group differences in change scores were evaluated using the Mann–Whitney U test. Categorical variables were analyzed using chi-square tests. To adjust for multiple testing, the Benjamini–Hochberg false discovery rate (FDR) correction was applied to the primary outcome family of acne lesion counts (total, inflammatory, and non-inflammatory) assessed across time points. All statistical tests were two-tailed, and a *p* value < 0.05 was considered statistically significant.

## 3. Results

### 3.1. Participant Characteristics

Of 135 volunteers who were screened for participation in the trial, 81 met the inclusion criteria and provided consent to participate in the supervised feeding trial for 20 weeks. As per the study protocol, the eligible participants (n = 81) were allocated to either almond (n = 40) or control groups (n = 41) using a computer-generated randomization method. In total, 7 participants were lost at follow-up and, the per protocol, analysis was performed in 74 participants (almond (n = 36) and control (n = 38)) who completed the trial and assessments at each of the time points from week 0 to week 20 (Figure 1).

The comparison of participant characteristics at baseline (week 0) showed no significant between-group differences in age, sex distribution, socioeconomic status, family history of cardiometabolic diseases, or the frequency or duration of acne episodes (Table 1). Acne severity was assessed using inflammatory, non-inflammatory, and total lesion counts and IGA grading of acne classification. The proportion of participants with mild (n = 56, almond (n = 28) and control (n= 28)) and moderate acne (n = 18, almond (n = 8), control (n = 10)) was comparable (*p* = 0.682) at baseline. Moreover, acne-related quality of life (ALQI), stress, anxiety, and sleep quality scores and energy intake did not show between-group differences.

### 3.2. Changes in Acne Count, Skin Morphology, and Facial Microflora Indices

Table 2 presents the pre- to post-intervention changes in acne lesion counts, acne morphology, facial hydration, and sebum measurements from week 0 to week 10 and week 20 in the almond group. The shift in facial skin microflora was measured using alpha diversity indices, such as Shannon diversity and the Chao1 index, from baseline (week 0) to endline (week 20). In the almond group, total acne lesion counts and non-inflammatory lesion counts demonstrated significant reductions at both midline and endline assessments compared with baseline based on unadjusted analyses (all unadjusted *p* < 0.05). After Benjamini–Hochberg FDR correction within the primary outcome family, reductions in total lesion counts remained statistically significant at midline (unadjusted *p* = 0.002; Benjamini–Hochberg FDR–adjusted *p* = 0.007) and at endline (unadjusted *p* < 0.001; adjusted *p* = 0.007). Similarly, non-inflammatory lesion counts were significantly reduced at midline (unadjusted *p* = 0.002; FDR-adjusted *p* = 0.007) and at endline (unadjusted *p* < 0.001; FDR-adjusted *p* = 0.007). The inflammatory lesion count decreased from week 0 to week 20 (adjusted *p* = 0.048). Objective lesion volume, area, and height measures for both single and clustered acne were also observed to significantly reduce by week 20 (*p* < 0.001). Facial photographs indicating changes in acne count and severity and sample reports of changes in morphometric characteristics of acne clusters at different time points from week 0 to week 20 are provided as Supplementary Files S1 and S2. 

Facial hydration levels improved at endline (*p* = 0.003), though the facial sebum measurements did not show statistically significant differences. Microbial alpha diversity indices increased significantly, with the Shannon index changing from 2.6 to 3.4 (*p* = 0.039) and the Chao1 richness index showing three-fold improvements (266.9 → 835.2; *p* < 0.001) at week 20. In the almond group, no statistically significant differences in acne lesion counts, acne volume, or facial hydration levels were observed in sex-stratified analyses.

### 3.3. Changes in Metabolic, Inflammatory, Anthropometric, and Psychosocial Outcomes

Significant post-intervention improvements in several cardiometabolic health parameters, anthropometry measurements, and lifestyle and psychosocial outcomes were observed in the almond group (Table 3). Total LDL cholesterol, non-HDL cholesterol, cholesterol: HDL cholesterol ratio, and fasting glucose levels showed significant reductions at endline. The inflammatory marker TNF-α concentrations decreased from baseline (83.2 (57.4–159.5) pg/mL) to endline (53.7 (16.7–92.1) pg/mL, *p* = 0.008), indicating improvements in systemic inflammation. Moreover, significant changes in psychosocial outcomes, such as acne-related quality of life scores (*p* < 0.001) and anxiety symptoms (*p* = 0.016), were observed at endline. Significant post-intervention gains in muscle mass and fat-free mass were also reported in the almond group.

### 3.4. Changes in Acne Lesion Count, Biochemical Markers, and Quality of Life Measures in Almond vs. Control Groups

**Figure 2:**
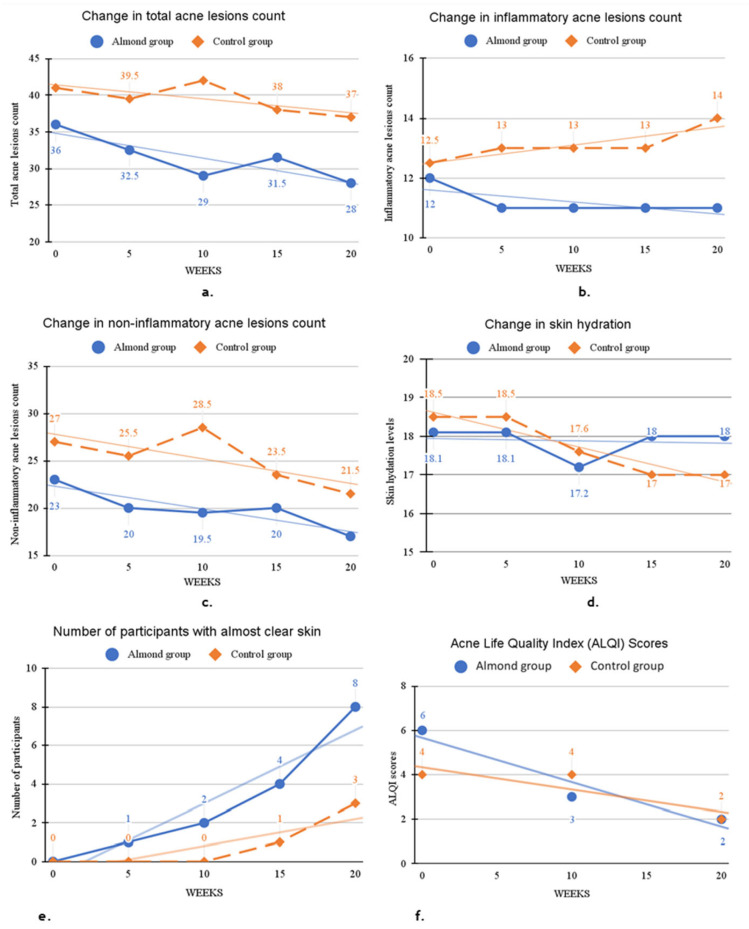
Changes in acne lesion count (**a**–**c**), facial skin hydration (**d**), and proportion of participants with clear skin and acne-related quality of life scores (**e**,**f**) from week 0 to week 20 in almond (n = 36) and control (n = 38) groups.

At week 20 (Table 4), the almond group showed greater reductions in total lesion counts (−22.2% vs. −9.8%), inflammatory lesion counts (−8.3% vs. +12%), and non-inflammatory lesion count (−26.1% vs. −20.4%) than controls. Significant between-group differences were observed for LDL cholesterol (*p* = 0.035) and ALQI scores (*p* = 0.047). Shannon– Weiner’s microbial diversity index increased in both groups, but the almond group exhibited larger gains (+30.8%, *p* = 0.039) compared to those in the control group (+12%, *p* =0.317). Changes in acne lesion counts, facial hydration levels, acne-related quality of life scores, and the proportion of participants with clear skin (IGA scores 0–1) were evaluated in the almond and control groups at multiple time points from week 0 to week 20 and are presented as graphs (a–f) in Figure 2.

To investigate the proportion of participants who transitioned from mild acne to minimal/no acne and from moderate acne to mild and minimal acne at week 20, we compared the changes in IGA grades of each participant in the almond and control groups. We observed that 25% (7/28) of almond group participants with mild acne at baseline had almost clear/clear skin (IGA scores 0–1) at week 20 compared to 10.7% (3/28) of control group participants. Additionally, pre–post assessments showed that 25% (7/28) of control group participants with mild acne at week 0 had worsened to moderate acne (IGA score 3) at week 20. For almond group participants with moderate acne at baseline (n = 8), 50% had improved to mild acne and 12.5% had almost clear skin (IGA score 1) post intervention.

## 4. Discussion

This study provides novel evidence that daily almond consumption is associated with clinically meaningful improvements in acne count and morphology, systemic inflammatory and metabolic health markers, facial skin microbial diversity, and acne-specific quality of life among young adults. These findings align with and extend emerging evidence linking diet, metabolic health, and acne pathophysiology [7,10,12,16]. The substantial reduction in non-inflammatory lesions and improvements in lesion morphology suggest that almonds may influence early comedogenesis, potentially through modulation of glycemic response, lipid metabolism, and oxidative stress. Almonds are rich in monounsaturated fats, vitamin E, zinc, and polyphenols—nutrients known to reduce oxidative stress and inflammatory cytokine production [23,45]. The observed decreases in acne lesion count and morphometric characteristics of single as well as cluster acne lesions, such as the volume, conforming area, and maximum height and inflammatory marker TNF-α, corroborate the therapeutic anti-inflammatory potential of almonds in skin health.

Results demonstrated an approximately 22% reduction in total acne lesion count following almond consumption, indicating a modest yet clinically meaningful improvement for individuals who prefer non-pharmacologic dietary approaches or complementary strategies alongside standard acne care. The observed favorable changes in fasting glucose and lipid profile in the almond group are consistent with prior trials that have reported regular almond consumption to blunt postprandial glycemia, improve lipid metabolism, and confer favorable cardiometabolic effects [24,25,26,46]. Existing mechanistic studies have demonstrated that the insulin/IGF-1 axis, sebum lipogenesis, and chronic inflammation are central to acne development. Hence, improvements in metabolic health markers concurrent with the decline in acne counts suggest a plausible metabolic route for the observed clinical benefits in the almond group participants.

In addition to the changes in skin and biochemical outcomes, we measured the alpha diversity of skin microflora using Chao1 and the Shannon index. The Shannon index evaluated alpha diversity in terms of total taxa and their relative abundances and Chao1 measured the estimated richness of a sample in terms of how many different taxa are present. Existing evidence highlights the central role of skin microflora in the pathogenesis of acne, particularly through its interaction with the pilosebaceous unit [47,48]. Specific strains of *Cutibacterium acnes*, a dominant resident of sebaceous regions, have been found to contribute to acne development by promoting inflammation, altering sebum composition, and disrupting follicular epithelial homeostasis [21,22,49]. These proinflammatory actions are mediated through keratinocyte proliferation, activation of Toll-like receptors, and release of cytokines that eventually lead to follicular obstruction and inflammatory lesion formation. Dysbiosis of the skin microbiome—characterized by reduced microbial diversity and an overrepresentation of pathogenic *C. acnes* strains—further amplifies oxidative stress, biofilm formation, and immune activation [22,50]. Together, these alterations in skin microflora can trigger and worsen acne by weakening skin barrier function and activating the cutaneous immune system.

While the gut–skin axis is well-established, diet-driven changes in the skin microbiome remain underexplored and represent an emerging research frontier [47]. Our study is among the few to examine how specific foods, such as almonds, influence skin microflora composition, which may in turn affect the onset, severity, and persistence of acne. The observed increase in both Chao1 values and Shannon diversity indices in the almond group at week 20 indicates a favorable shift in microbial diversity and richness. Within-group analyses showed a 30.8% improvement in the almond group compared with a 12.0% improvement in the control group post intervention. Although between-group differences did not meet statistical significance, the improvements in microbial alpha diversity suggest a directional trend that can be considered biologically relevant and attributable to the systemic or nutrient-mediated modulatory effects of almond consumption on the skin’s ecological environment and host–microbe interactions. In previous studies, higher skin microbial diversity has been linked to improved epidermal barrier function and reduced inflammatory potential [21,49], providing a plausible mechanism for the observed improvements in lesion counts in our study. Yet, the current findings should be viewed as preliminary evidence that supports associations and potential mechanisms rather than definitive causal pathways between almond consumption, skin microflora, and the gut–skin axis.

Mechanistically, these outcomes can be attributed to almonds’ dietary fiber, unsaturated fat, and polyphenol content, which have been reported to increase butyrate-producing genera, such as *Roseburia* and *Lachnospira*, in the gut [51,52]. A recent study showed that both whole almonds and almond skins can exert prebiotic effects, significantly increasing *Bifidobacterium* and *Lactobacillus* [53]. Although these studies investigated the gut microbiome rather than skin, they provide biological plausibility that almond consumption enriches beneficial microbial taxa and increases microbial diversity through the bidirectional gut–skin axis. Alterations in gut microbiota, such as changes in diversity, short-chain fatty acid production, or systemic inflammatory mediators, can influence skin barrier integrity, immune responses, and sebum regulation, thereby affecting overall skin health and acne outcomes.

We observed improvements in anxiety symptoms and acne-related quality of life scores post almond supplementation. However, sleep quality assessed using the PSQI scale scores showed an increase, indicating a worsening of sleep patterns in the almond group. The existing literature highlights the complexity of the diet–sleep relationship, as several factors, including overall dietary pattern, timing of food intake (chrono nutrition), individual metabolic differences, and lifestyle behaviors, such as screen time and physical activity, can mediate the influence of diet on both sleep quantity and duration [54]. Systematic reviews suggest that healthier dietary patterns, such as diets rich in fruits, vegetables, and sources of tryptophan or melatonin, tend to be associated with better self-reported sleep quality, whereas diets high in processed or free-sugar-rich foods are negatively associated with sleep measures [55,56]. In a multi-component lifestyle intervention that included dietary change, there were no statistically significant between-group differences in PSQI-determined sleep quality outcomes after 6 and 12 months despite changes in diet and physical activity components [57]. Another scoping review of 33 studies examining Mediterranean dietary patterns and sleep observed inconsistent results, with cross-sectional analyses showing positive influences on sleep and two of the three selected trials reporting no significant improvements in sleep quality post intervention [58]. Given that the existing evidence is limited by heterogeneous study designs and does not establish a causal relationship, we recommend cautious interpretation of our findings and emphasize the need for further investigation in future studies that are specifically designed to assess sleep outcomes using both subjective and objective measures.

Collectively, the concordant improvements across lesion counts, lesion morphology, systemic biomarkers, microbial diversity, and quality of life in the almond group strengthen the causal inference beyond isolated clinical changes. The greater reduction in non-inflammatory compared to inflammatory lesions suggests that almonds may have a stronger impact on early comedonal processes—such as keratinocyte proliferation, sebum composition, and glycemia-related mechanisms—than on established inflammatory pathways. These preliminary results, however, warrant further investigations in longer trials or studies that incorporate dietary interventions as adjunctive treatments of adult acne. The marked improvements in acne-related quality of life and composite anxiety scores parallel the reductions in lesion counts and morphology, indicating that clinical gains could have translated into meaningful psychosocial benefits for the almond group participants.

While the results are encouraging, our study has certain limitations that must be considered when interpreting the outcomes. The modest sample size may limit statistical power for the subgroup analyses, and the 20-week trial duration could capture medium-term effects but not long-term sustainability. The skin microbiome analyses were conducted in a subsample of participants due to logistical and financial constraints associated with sample collection, sequencing, and bioinformatic processing. Although the study sample size was determined based on a priori power calculations for the primary clinical outcomes (acne lesion counts and skin biophysical measures) and align with previous food-based intervention studies examining skin health and related skin microbiome outcomes [59,60], particularly in early-phase or exploratory trials, the current findings should be viewed as preliminary evidence, warranting further investigations in larger sample sets. Future studies may also consider a mixed-effects modeling approach to more comprehensively evaluate group × time interactions and longitudinal intervention effects. Randomized controlled trials with stratified analyses by sex, hormonal status, and baseline metabolic profile would help confirm the findings of our study and identify responders. Mechanistic studies that combine metagenomic/functional microbiome profiling, sebum lipidomics, and hormone/IGF-1 axis assays will help map pathways connecting almond intake to the observed cutaneous effects. Implementation research assessing the acceptability, affordability, and integration of nut-based dietary advice into acne care pathways can also be considered in the future to inform public health communication strategies.

Despite these limitations, our study has several strengths. A notable strength is the multidimensional outcome assessment that integrated clinical acne grading, objective lesion morphometry, biochemical markers, skin microbiome diversity metrics, and patient-reported quality of life to evaluate the effectiveness of almond supplementation in acne. The prospective pre–post design with a parallel control group to draw within- and between-group comparisons adds to the robustness of the study design and strengthens the internal validity of the study. While participant blinding was not feasible due to the nature of the dietary intervention, the study was investigator-blinded, and the investigators and laboratory personnel involved in outcome assessment, data handling, and statistical analysis remained blinded to product allocation throughout the study period. The self-reported measures, such as acne-related quality of life, perceived stress, anxiety, and sleep, were assessed using validated questionnaires administered in a standardized manner across groups and time points. To mitigate expectancy and performance bias, participants in both groups received equivalent study contact and similar levels of monitoring (including video calls) to maintain compliance and data integrity. Another strength of the study was that unlike most acne trials that have been conducted among adolescent groups or in clinical settings, we enrolled community-living young adults—a population that remains underrepresented in acne research, thereby contributing to the real-world evidence of our findings. Finally, to our knowledge, this is the first study to investigate the effect of almond consumption on acne outcomes and to relate these changes to shifts in skin microflora composition.

## 5. Conclusions

In conclusion, this study demonstrates the potential of a simple, scalable, food-based strategy to support skin health while also conferring cardiometabolic benefits. Given the rising prevalence of adult acne and the growing interest in lifestyle-based, non-pharmacological management approaches, the findings underscore the potential of almonds as a nutrient-dense snacking option that can complement standard acne treatments with long-term benefits for both skin and metabolic health. The observed consistency of improvements across outcomes shows that almonds may exert multi-system benefits relevant to acne, highlighting a promising avenue for future research to explore food–microbiome–skin interactions in adult acne.

## Figures and Tables

**Table 1:** Participant characteristics at baseline.

	Almond Group (n = 36)	Control Group (n = 38)	*p* Value
Sex, n (%)			
Male	17 (47.2)	19 (50.0)	0.811
Female	19 (52.8)	19 (50.0)	
Socioeconomic status, n (%)			
Upper (26–29)	2.0 (5.6)	2 (5.3)	0.736
Upper middle (16–25)/lower middle (11–15)	28.0 (77.8)	32 (84.2)	
Upper lower (5–10)/lower (<5)	6.0 (16.7)	4 (10.5)	
Regular menstrual cycle, n (%)			
21–35 days	18 (94.7)	17 (89.4)	0.808
Acne lesion counts, Median (IQR)			
Inflammatory acne lesion count	12.0 (11.0–15.2)	12.5 (10.0–17.0)	0.656
Non-inflammatory acne lesion count	23.0 (17.5–31.0)	27.0 (20.2–35.8)	0.150
Total acne lesion count	36.0 (29.0–47.0)	41.0 (33.2–52.0)	0.113
Acne severity as per IGA, n (%)			
Mild acne (IGA = 2)	28 (77.8)	28 (73.7)	0.682
Moderate acne (IGA = 3)	8 (22.2)	10 (26.3)	
Acne episode frequency, n (%)			
Daily	1 (2.9)	1 (2.6)	0.346
Once every 2–3 days	6 (17.1)	13 (34.2)	
Once a week	10 (28.6)	11 (28.9)	
Less than once a week	18 (51.4)	13 (34.2)	
Duration of acne episodes, n (%)			
≤1 week	15 (42.9)	18 (47.4)	0.882
1–2 weeks	10 (28.6)	9 (23.7)	
>2 weeks	10 (28.6)	11 (28.9)	
Acne quality of life, Median (IQR)	6.0 (3.0–10.2)	4.0 (2.0–6.8)	0.065
Anthropometry and lifestyle, Median (IQR)			
Body mass index (kg/m^2^)	23.2 (20.3–26.4)	23. 2 (20.2–26.1)	0.753
Stress (PSS-10 scores)	18.0 (16.0–24.2)	20.0 (16.2–25.0)	0.475
Anxiety (GAD-7 scores)	14.5 (10.8–18.2)	14.5 (12.0–20.0)	0.551
Sleep quality (PSQI scores)	3.5 (2.0–6.2)	4.0 (1.2–6.8)	0.926
Energy intake (Kcal)	2073.0 (1490.8–2505.3)	1937.5(1535.0–2699.3)	0.863

IQR, Intra Quartile Range. IGA, Investigator Global Assessment. PSS, Perceived Stress Scale. GAD, Generalized Anxiety Disorder. PSQI, Pittsburg Sleep Quality Index. Socioeconomic status classified using Modified Kuppuswamy scale, India.

**Table 2:** Changes in acne count, skin microflora, and quality of life scores from baseline (week 0) to midline (week 10) and endline (week 20) in almond group (n = 36).

Variables	Week 0 Median (IQR)	Week 10 Median (IQR)	*p* Value ^#^	Week 20 Median (IQR)	*p* Value ^^^
Inflammatory acne lesion count	12.0 (11.0–15.2)	11.0 (8.0–14.0)	0.135 ^†^	11.0 (5.5–14.0)	0.048 *^†^
Non-inflammatory acne lesion count	23.0 (17.5–31.0)	19.5 (14.0–23.3)	0.007 *^†^	17.0 (14.0–24.2)	<0.007 *^†^
Total lesion acne count	36.0 (29.0–47.0)	29.0 (22.0–37.3)	0.007 *^†^	28.0 (20.0–35.8)	<0.007 *^†^
Single Acne					
Volume of acne (mm^3^)	2.7 (1.3–4.2)	1.0 (0.1–2.5)	<0.001 **	0.7 (0.1–2.7)	<0.001 **
Conforming area (mm^2^)	21.7 (10.1–59.3)	11.4 (1.8–39.1)	<0.001 **	8.0 (2.0–45.0)	<0.001 **
Maximum height of acne (mm)	0.3 (0.2–0.3)	0.1 (0.1–0.2)	<0.001 *	0.1 (0.1–0.2)	<0.001 **
Cluster Acne					
Volume of acne (mm^3^)	2.2 (0.9–7.5)	0.8 (0.1–4.3)	<0.001 **	0.9 (0.1–2.5)	<0.001 **
Conforming area (mm^2^)	20.0 (9.6–83.4)	10.8 (1.7–57.2)	<0.001 **	9.9 (2.7–39.5)	<0.001 **
Maximum height of acne (mm)	0.3 (0.2–0.3)	0.1 (0.1–0.2)	<0.001 **	0.1 (0.1–0.2)	<0.001 **
Facial sebum measurement (µg)	22.0 (12.0–30.2)	26.0 (15.0–32.3)	0.302	22.0 (15.5–29.5)	0.562
Facial hydration (AU)	18.1 (10.9–28.3)	17.2 (12.2–28.7)	0.305	18.1 (13.4–30.7)	0.003 *
Alpha Diversity Indices of Facial Skin Microflora					
Shannon–Wiener index	2.6 (2.1–2.8)	-	-	3.4 (2.3–5.1)	0.039 *
Chao1 index	266.9 (126.1–548.4)	-	-	835.2 (435.0–1332.8)	<0.001 **
Total no. of genera	77 (20.0–106.5)	-	-	141 (94.8–231.3)	<0.001 **
Acne quality of life (ALQI scores)	6.0 (3.0–10.2)	3.0 (1.0–5.0)	0.011 *	2.0 (0.0–4.2)	<0.001 **

^^^ Non-parametric repeated-measures ANOVA (Friedman test). ^†^ Adjusted *p* value using Benjamini–Hochberg Correction. ^#^ Wilcoxon-signed rank test. * *p* < 0.05, ** *p* < 0.001.

**Table 3:** Changes in biochemical, anthropometric, stress, and sleep quality from baseline (week 0) to midline (week 10) and endline (week 20) in almond group (n = 36).

Variables	Week 0 Median (IQR)	Week 10 Median (IQR)	*p* Value ^#^	Week 20 Median (IQR)	*p* Value ^^^
LDL Cholesterol (mg/dL)	100.3 (78.3–119.2)	91.7 (77.8–119.6)	0.358	88.4 (66.2–103.8)	0.048 *
Cholesterol: HDL-C ratio	3.7 (3.1–4.1)	3.4 (3.2–3.9)	0.177	3.3 (3–3.6)	0.005 *
Non-HDL Cholesterol	121.6 (93.0–132.0)	104.2 (91.7–132.3)	0.291	103.6 (79.3–121.8)	0.003 *
Fasting Glucose (mg/dL)	84.0 (79.5–89.0)	82.0 (77.0–86.3)	0.138	79.0 (74.5–85.0)	0.007 *
Fasting Insulin (uIU/mL)	7.9 (5.6–10.5)	7.9 (5.7–11.4)	0.730	7.1 (6.0–8.8)	0.920
Creatinine (mg/dL)	0.7 (0.6–0.8)	0.7 (0.5–0.8)	0.944	0.6 (0.5–0.8)	0.044 *
TNF-alpha (pg/mL)	83.2 (57.4–159.5)	-	-	53.7 (16.7–92.1)	0.008 *
IL6 (pg/mL)	0.1 (0.1–1.4)	-	-	1.2 (0.1–2.1)	0.117
BMI (kg/m^2^)	23.2 (20.3–26.4)	23.4 (20.6–26.6)	0.309	23.2 (20.5–26.8)	0.238
Body Fat %	27.6 (20.1–39.3)	29.6 (21.4–39.6)	0.355	28.0 (21.5–39.2)	0.276
Muscle Mass (kg)	40.5 (34.6–46.0)	41.0 (34.7–46.7)	0.214	40.9 (34.6–46.4)	0.012 *
Fat-Free Mass (kg)	42.9 (36.6–48.5)	43.2 (36.8–49.3)	0.197	43.2 (36.7–49.0)	0.010 *
Stress (PSS-10 scores)	18.0 (16.0–24.3)	-	-	17.5 (16.0–21.0)	0.056
Anxiety (GAD-7 scores)	14.5 (10.8–18.3)	-	-	13.0 (10.0–15.0)	0.016 *
Sleep Quality (PSQI scores)	3.5 (2.0–6.3)	-	-	9.5 (4.8–11.0)	<0.001 **

^^^ Non-parametric repeated-measures ANOVA (Friedman test). ^#^ Wilcoxon-signed rank test. * *p* < 0.05, ** *p* < 0.001.

**Table 4:** Comparison of changes in acne and skin characteristics, biochemical parameters, and QoL between the almond and control groups.

	Almond Group (n = 36)			Control Group (n = 38)			*p* Value ^#^
	Week 0 Median (IQR)	Week 20 Median (IQR)	%Change, *p* Value ^^^	Week 0 Median (IQR)	Week 20 Median (IQR)	%Change, *p* Value ^^^	
Inflammatory Acne Lesion	12.0 (11.0–15.2)	11.0 (5.5–14.0)	−8.3%, 0.048 *^†^	12.5 (10.0–17.0)	14.0 (9.0–16.0)	12%, 0.912 ^†^	0.090 ^†^
Non-Inflammatory Acne Lesion Count	23.0 (17.5–31.0)	17.0 (14.0–24.2)	−26.1%, 0.007 *^†^	27.0 (20.2–35.8)	21.5 (16.0–28.0)	−20.4%, 0.009 *^†^	0.548 ^†^
Total Acne Lesion Count	36 (29–47)	28 (20–35.8)	−22.2%, 0.007 *^†^	41 (33.2–52)	37 (26.2–42.5)	−9.8%, 0.060 ^†^	0.195 ^†^
Facial Sebum Content (µg)	22.0 (12.0–30.2)	22.0 (15.5–29.5)	0%, 0.562	19.0 (11.5–26.0)	20.5 (14.0–25.0)	7.9%, 0.785	0.820
Facial Hydration Levels (AU)	18.1 (10.9–28.3)	18.1 (13.4–30.7)	0%, 0.003 **	18.5 (13.5–26.5)	17.0 (11.2–27.7)	−8.1%, 0.871	0.102
Shannon Diversity Index	2.6 (2.1–2.8)	3.4 (2.3–5.1)	+30.8%, 0.039 *	2.5 (1.3–3.2)	2.8 (1.3–3.5)	+12%, 0.317	0.595
Chao1 Diversity Index	266.9 (126.1–548.4)	835.2 (435.0–1332.8)	+219.3%, <0.001 **	223.0 (181.5–287.1)	554.3 (436.6–840.8)	+148.5%, 0.317	0.263
TNF-Alpha (pg/mL)	83.2 (57.4–159.5)	53.7 (16.7–92.1)	−35.5%, 0.008 *	95.3 (62.5–197.3)	69.8 (24.3–118.3)	−26.8%, 0.002 *	0.480
IL6 (pg/mL)	0.1 (0.1–1.4)	1.2 (0.1–2.1)	+110%, 0.117	1.2 (0.1–1.9)	1.3 (1.0–1.9)	+8.3%, 0.732	0.109
Acne Quality of Life	6.0 (3.0–10.2)	2.0 (0.0–4.2)	−66.7%, <0.001 **	4.0 (2.0–6.8)	2.0 (0.2–7.0)	−50%, 0.628	0.047 *

IQR, interquartile range. TNF, Tumor Necrosis Factor. IL6, Interleukin-6. * *p* <0.05: ** *p* <0.001. ^†^ Adjusted *p* value using Benjamini–Hochberg Correction. ^^^ Non-parametric repeated-measures ANOVA (Friedman test). ^#^ Mann–Whitney U test.

## Data Availability

All data generated and/or analyzed during the current study are included in this published article and the Supplementary Materials. Additional information is available from the corresponding author upon reasonable request and subject to relevant ethical and institutional approvals.

## Supplementary Materials

The following supporting information can be downloaded at https://www.mdpi.com/article/10.3390/nu18040625/s1, File S1: Facial photographs indicating changes in acne count and severity from week 0 to week 20 in the almond group; File S2: Sample reports of changes in morphometric characteristics of acne clusters at different time points from week 0 to week 20.
