# Supplementary material for: Almond Supplementation Improves Acne Lesions and Skin Microbial Diversity in Adults with Mild to Moderate Acne Vulgaris
Source: Nutrients. 2026 Feb 13;18(4):625. doi: 10.3390/nu18040625 (PMC12943583; doi:10.3390/nu18040625)
Supplement: Supplementary file 1 [file nutrients-18-00625-s001.zip › Supplementary File S1.pdf]

Supplementary File S1: Facial photographs indicating changes in acne count and severity from week 0 to week 20 in the almond group

Supplementary File S1A. Facial photographs taken at different angles of a participant for representation

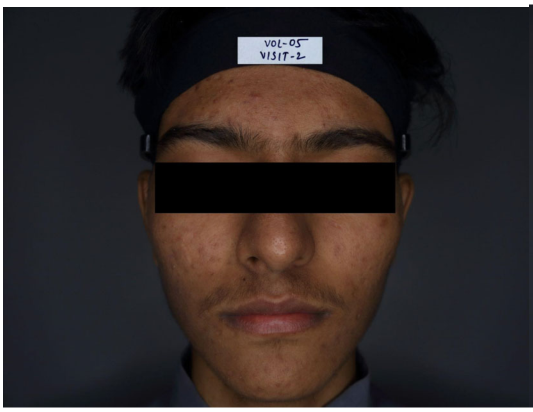

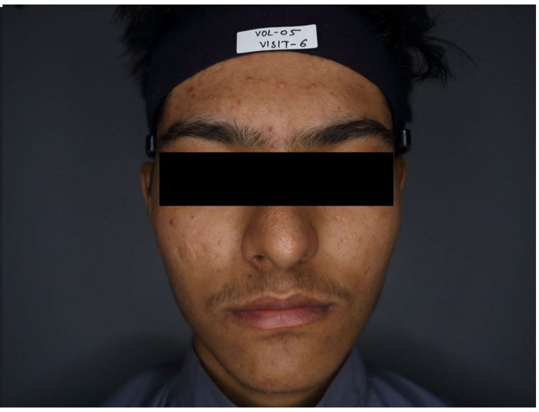

A B

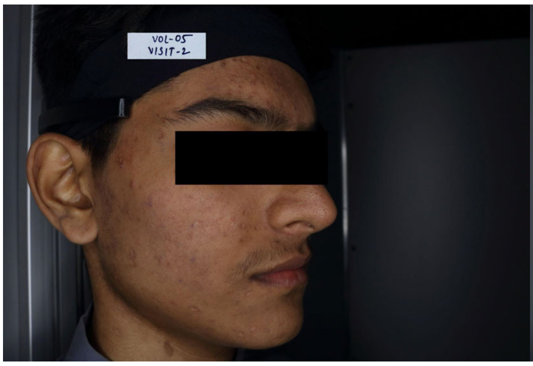

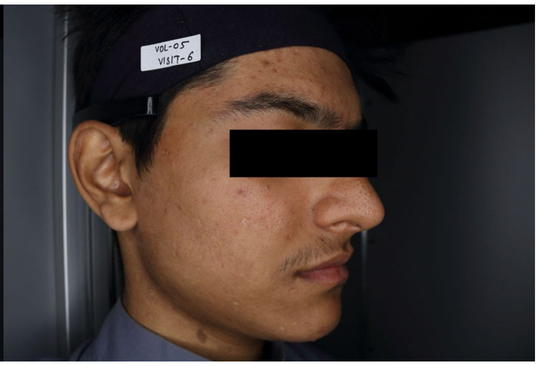

C D

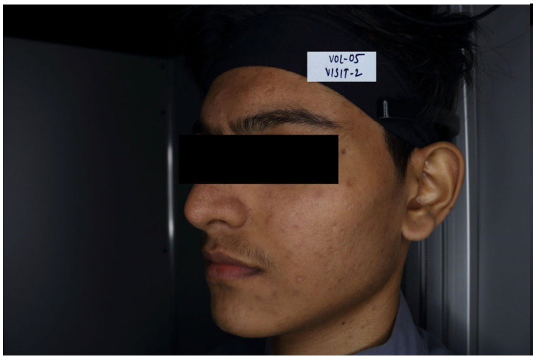

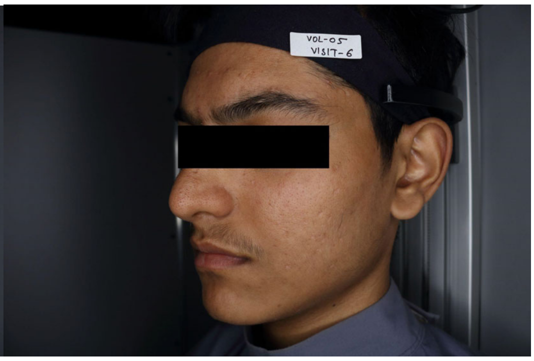

E F

A and B: front view (90°); C and D: left-side view (0°);

E and F: right-side view (180°) of participant no. 05 at week-0 and week-12

Supplementary File S1B: Right-side view (180°) facial photographs of acne in three almond group participants from week 0 to week 20

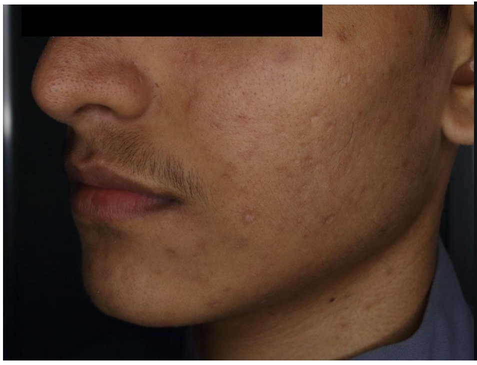

A

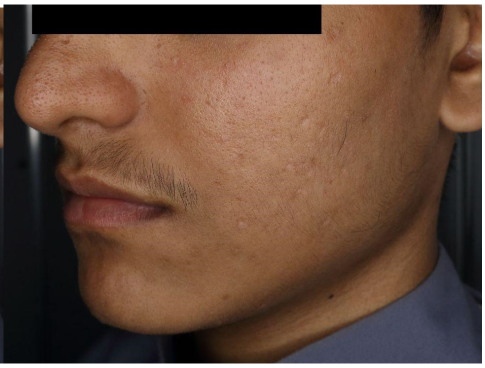

B

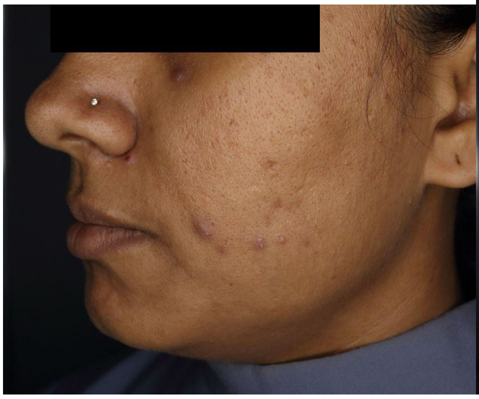

C

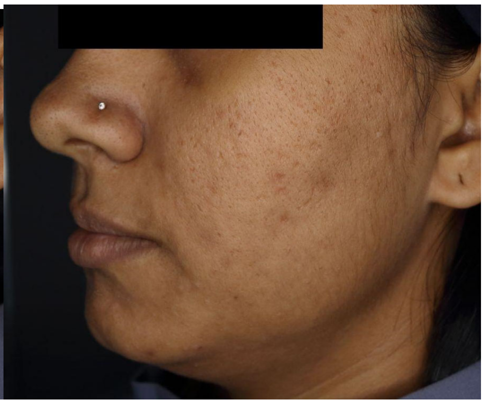

D

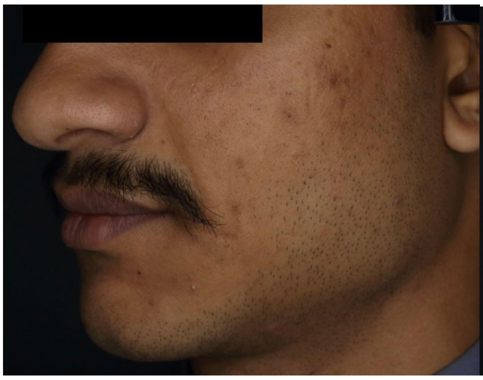

E

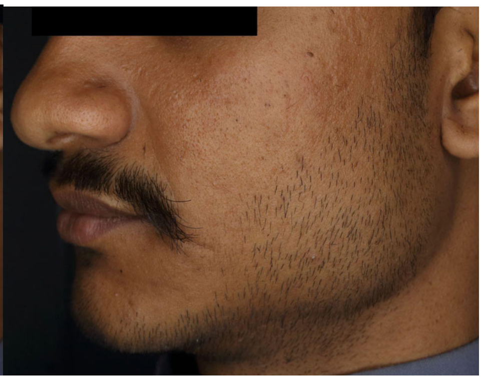

F

A and B: Participant 1; C and D: Participant 2; E and F: Participant 3, at week-0 and week-12
