# Supplementary material for: Almond Supplementation Improves Acne Lesions and Skin Microbial Diversity in Adults with Mild to Moderate Acne Vulgaris
Source: Nutrients. 2026 Feb 13;18(4):625. doi: 10.3390/nu18040625 (PMC12943583; doi:10.3390/nu18040625)
Supplement: Supplementary file 1 [file nutrients-18-00625-s001.zip › Supplementary File S2.pdf]

Supplementary File S2: Sample Reports of changes in morphometric characteristics of acne clusters at different time points from week 0 to week 20 using Antera ® 3D imaging in almond and control groups

Supplementary File S2A*:* Changes in acne clusters from week 0 to week 20 in a participant with mild acne in the almond group

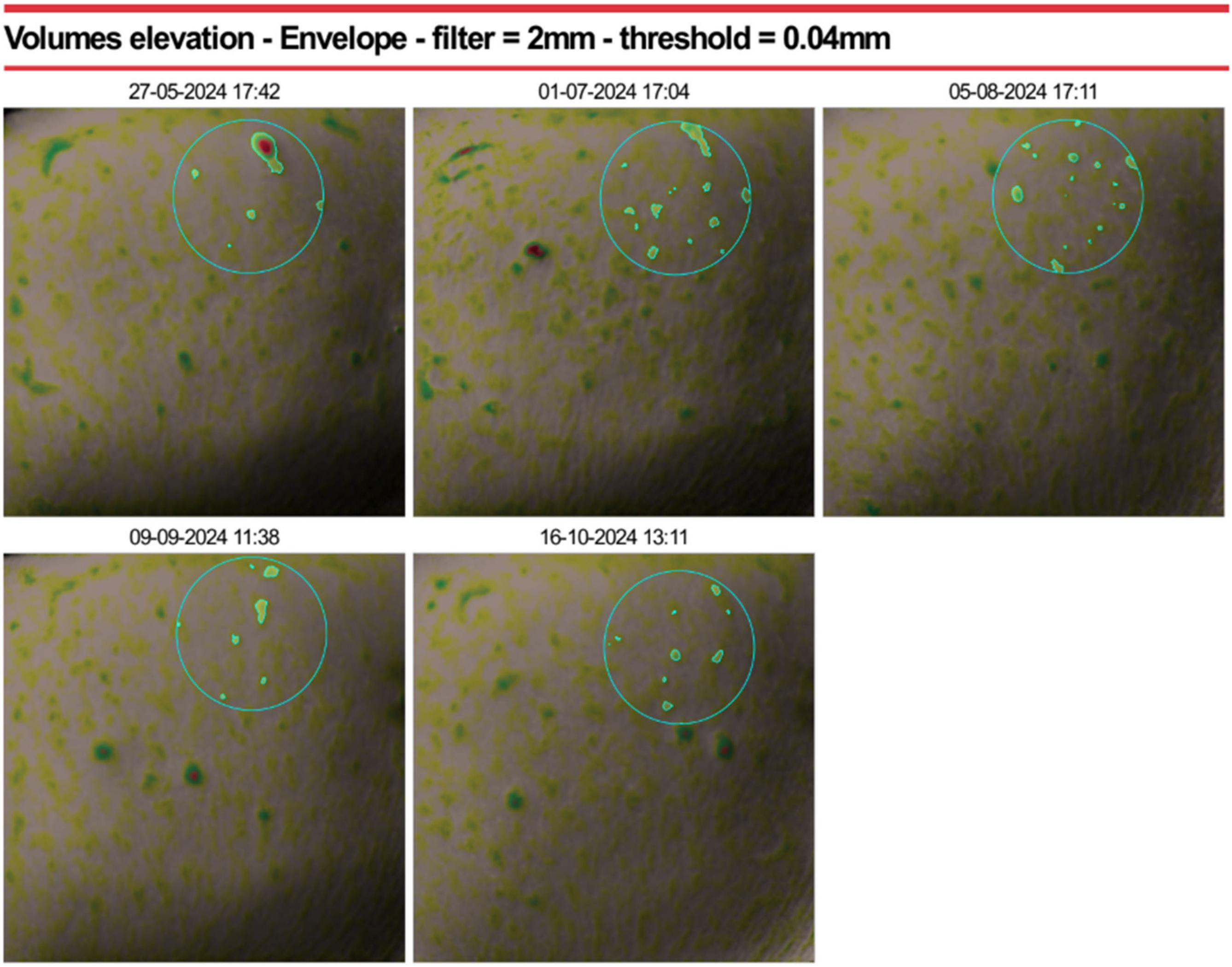

Feature	27-05-2024	35 days	70 days	105 days	142 days
Volume [mm³]	1.45	0.91	0.59	0.42	0.33
Conforming area [mm²]	16.18	17.12	11.08	8.33	6.6
Maximum height [mm]	0.23	0.1	0.1	0.07	0.09
Mean height [mm]	0.09	0.053	0.054	0.05	0.051

Supplementary File S2B- Changes in acne clusters from week 0 to week 20 in a participant with mild acne in the control group

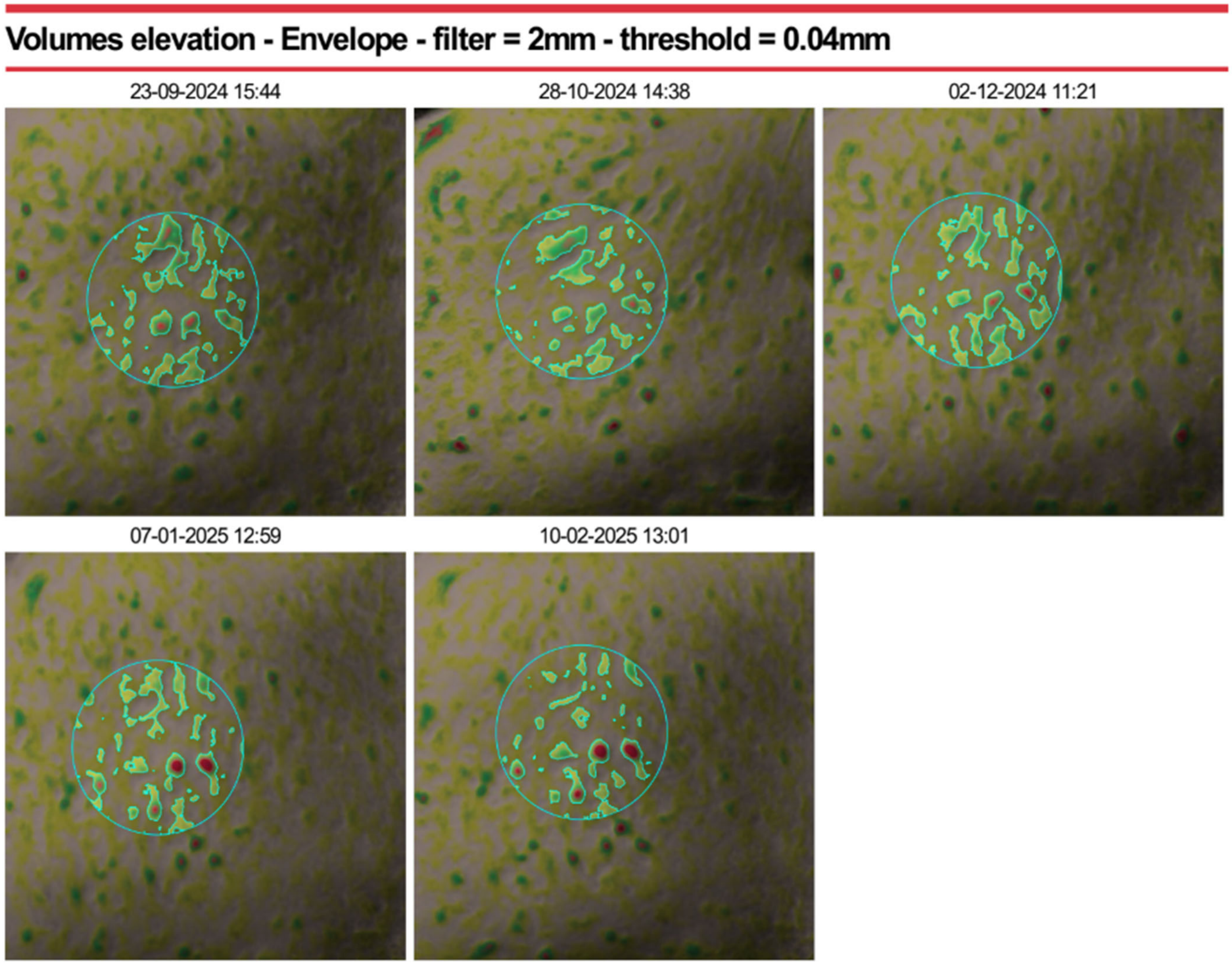

Feature	23-09-2024	35 days	70 days	106 days	140 days
Volume [mm^3]	6.94	6.37	7.17	6.5	5.88
Conforming area [mm^2]	108.58	97.69	115.31	96.4	78.69
Maximum height [mm]	0.17	0.16	0.18	0.27	0.29
Mean height [mm]	0.064	0.065	0.062	0.067	0.075

Supplementary File S2C: Changes in acne clusters from week 0 to week 20 in a participant with moderate acne in the almond group

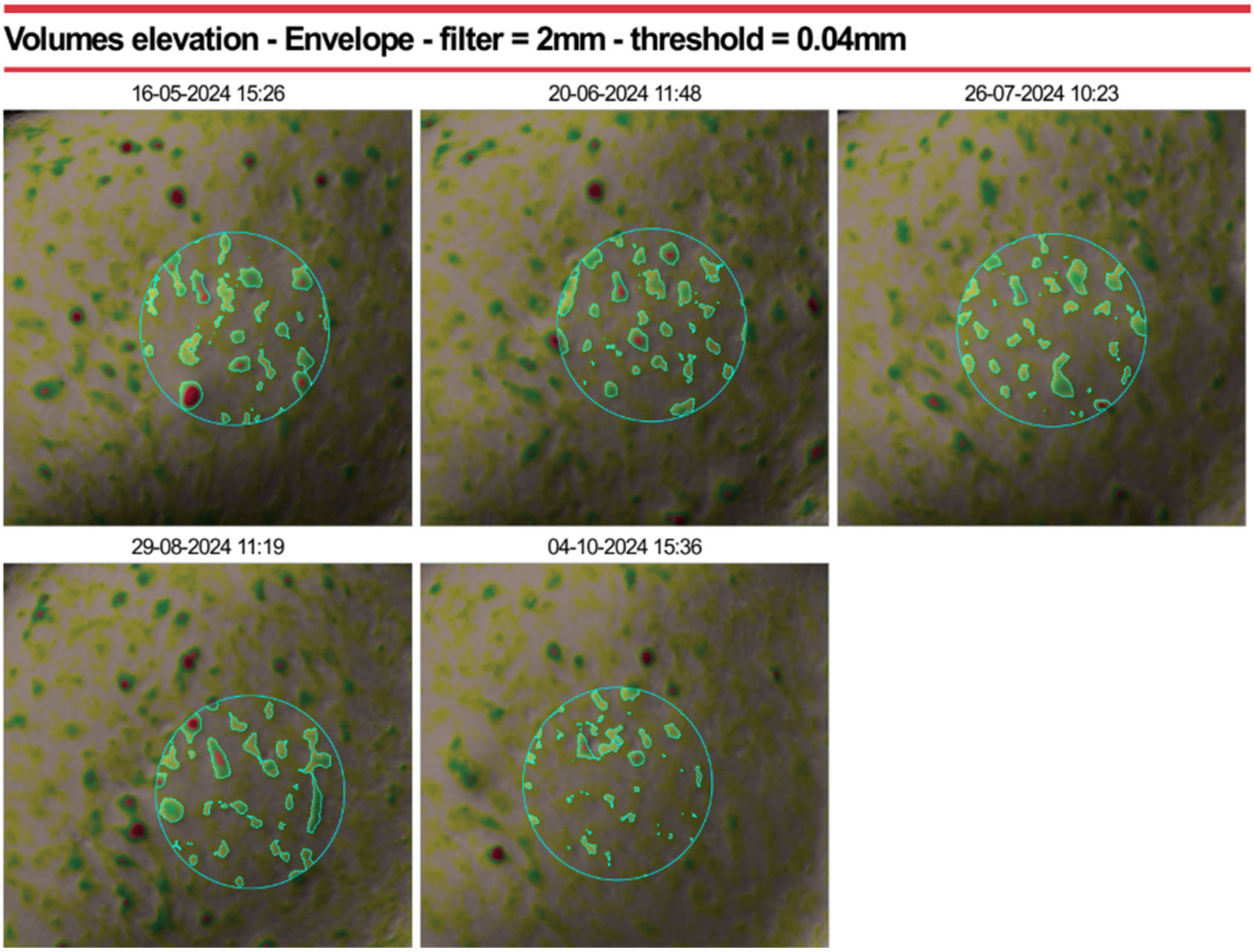

Feature	16-05-2024	35 days	71 days	105 days	141 days
Volume [mm³]	7.44	5.95	5.15	6.15	2.55
Conforming area [mm²]	97.46	90.19	82.65	91.01	45.55
Maximum height [mm]	0.34	0.18	0.21	0.24	0.16
Mean height [mm]	0.076	0.066	0.062	0.068	0.056

Supplementary File S2D: Changes in acne clusters from week 0 to week 20 in a participant with moderate acne in the control group

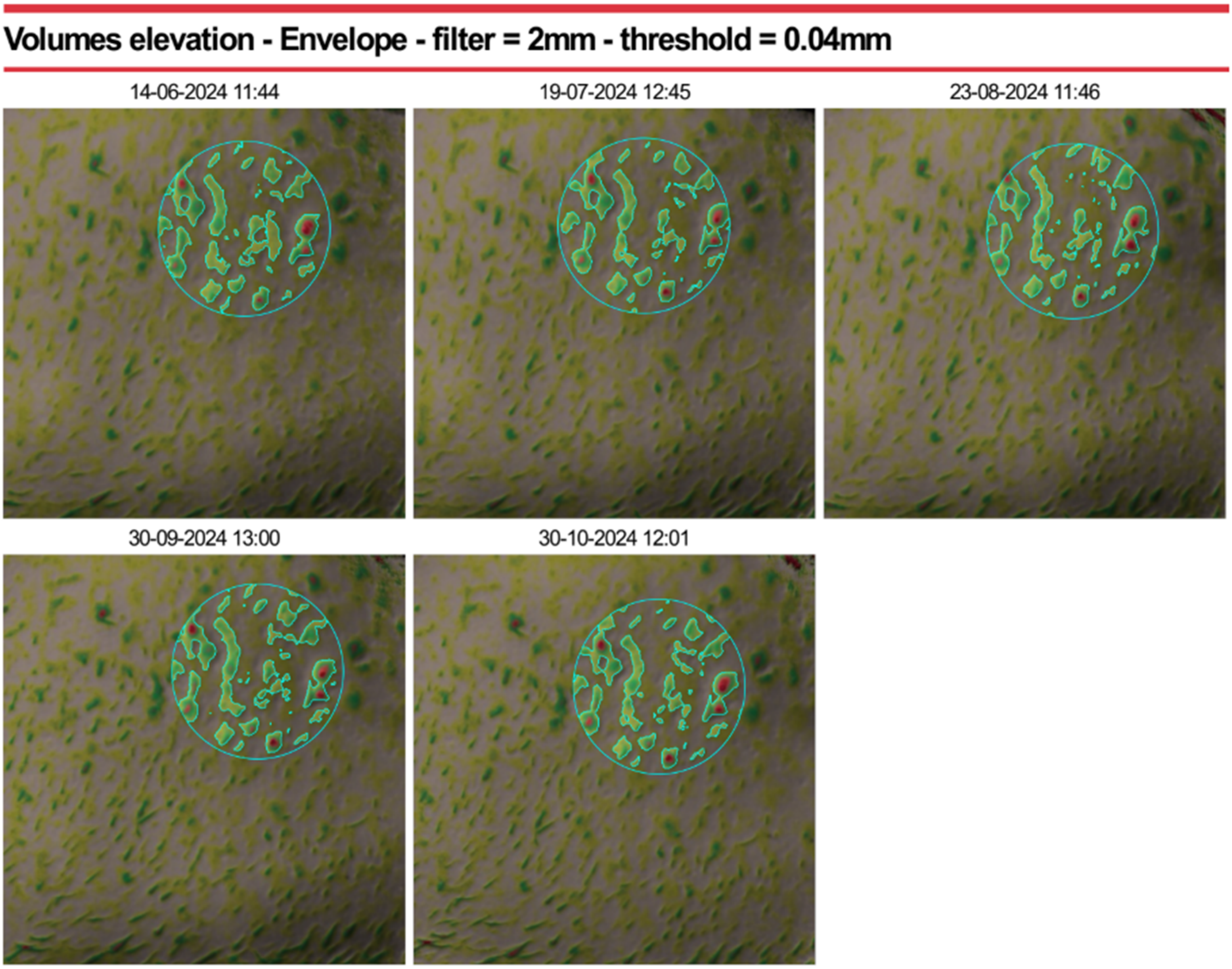

Feature	14-06-2024	35 days	70 days	108 days	138 days
Volume [mm³]	8.9	8.51	8.53	9.34	9
Conforming area [mm²]	129.22	120.04	119.51	129.64	125.45
Maximum height [mm]	0.22	0.22	0.22	0.22	0.23
Mean height [mm]	0.069	0.071	0.071	0.072	0.072
